# Whole exome sequencing identifies common mutational landscape of cervix and endometrium small cell neuroendocrine carcinoma

**DOI:** 10.3389/fonc.2023.1182029

**Published:** 2023-10-18

**Authors:** Wei Wang, Fan Zhang, Yan Li, Bo Chen, Yu Gu, Ying Shan, Yaping Li, Wei Chen, Ying Jin, Lingya Pan

**Affiliations:** ^1^Department of Obstetrics and Gynecology, Peking Union Medical College Hospital, Chinese Academy of Medical Sciences and Peking Union Medical College, National Clinical Research Center for Obstetric and Gynecologic Diseases, Beijing, China; ^2^Department of Obstetrics and Gynecology, The Fifth People’s Hospital of Ningxia, Shizuishan, China; ^3^Beijing Advanced Innovation Centre for Biomedical Engineering, Key Laboratory for Biomechanics and Mechanobiology of Ministry of Education, School of Engineering Medicine, Beihang University, Beijing, China; ^4^Department of Pathology, Peking Union Medical College Hospital, Chinese Academy of Medical Sciences and Peking Union Medical College, Beijing, China

**Keywords:** small cell neuroendocrine carcinoma of cervix, small cell neuroendocrine carcinoma of endometrium, whole-exome sequencing, mutational signatures, PI3K

## Abstract

**Background:**

Primary small cell neuroendocrine carcinomas of the cervix and endometrium are rare gynecological malignancies with limited treatment options. This study aimed to improve the understanding of the carcinogenesis process and identify potential therapeutic targets for these two tumor types by constructing the mutational landscape at the whole exome level.

**Methods:**

Primary tumor tissues and their matched blood samples were obtained from 10 patients with small cell cervical neuroendocrine carcinoma (NECC) and five patients with small cell endometrial neuroendocrine carcinoma (NECE). Whole exome sequencing was performed to construct the somatic mutation profiles. Mutational signature and recurrent mutated gene analysis were used to identify tumor subtypes and common carcinogenesis processes.

**Results:**

Based on the burden of different mutational signatures, the NECCs in this work can be divided into two subtypes, including the mismatch repair deficiency like (dMMR-like) type (4/10) and the high spontaneous deamination type (6/10). Components of the PI3K/AKT signaling and RAS signaling were exclusively mutated in these two subtypes, respectively. The integration of human papillomavirus made a limited contribution to tumorigenesis in NECC (20%). The dysfunction of the mismatch repair system and microsatellite instability are the major features of NECE. PI3K/AKT, JAK/STAT signaling, and chromatin remodeling activity were the common mutated pathways in NECE. *PIK3CA, WNK2*, and *KMT2B* underwent mutations in both the dMMR-like subtype of NECC (50% – 75%) and in NECE (60% – 80%) specimens, while exhibiting infrequent mutational occurrences in publicly available data pertaining to neuroendocrine carcinomas of the lung or bladder (< 10%).

**Conclusion:**

We identified the two subtypes of NECC with distinct mutated pathways and potential therapy targets. The dMMR-like type NECC and NECE may share a similar carcinogenesis process that include dysfunction of PI3K/AKT signaling, cell cycle, antiapoptotic processes, and chromatin remodeling activity.

## Introduction

Neuroendocrine carcinomas (NEC) usually occur in the intestine, pancreas, or lung and have characteristic histological and immunohistochemical features (1–3). A report on female genital tumors revised by the World Health Organization in 2020 describes neuroendocrine carcinomas as poorly differentiated neoplasms, dividing them into three categories, namely small cell, large cell, and admixed neuroendocrine carcinomas (4). Neuroendocrine carcinomas originating from the female genital tract including the endometrium and cervix are relatively rare (5). Due to the small number of cases, current research content and treatment recommendations are usually extrapolated from histologically similar neuroendocrine carcinomas in other organs or based on previous retrospective studies without large-scale sequencing analyses, which does not allow a comprehensive understanding of their genetic characteristics or allow guiding of clinical treatment strategies (6).

To date, there are limited data on whole exome sequencing (WES) for small cell neuroendocrine carcinomas of the endometrium and cervix, and there is especially a lack of data from Chinese patients, which may reflect the rarity of this type of disease. Several published studies have analyzed the mutational landscape of small cell neuroendocrine carcinoma of the cervix (NECC). Commonly mutated genes include *TP53, KRAS, PIK3CA, c-Myc*, *KTM2D*, and *PTEN* (6–11), and homologous recombination repair mutation genes include *ATM*, *PALB2*, *FANCA*, *FANCL*, and *FANCF* (8). There are deletions of the tumor suppressor gene *LATS1* and amplification of *MYC*, *IRS2*, *TERT*, *IL17R*, *RICTOR*, *CDK8*, *SOX2*, *BRCA2*, and other genes in the study (6, 8). Cho et al. applied WES to NECC, identified recurrent mutations of *ATRX* and *ERBB4*, and proposed that the NECC mutation spectrum is characterized by a predominant C > T/G > A transition (7). Hillman et al. performed WES on 15 NECC samples and found that one tumor exhibited a somatic mutation rate more than ten times that of the median for the cohort and that the tumor also contained the pathogenic *MSH2* missense mutation (p.G164R) (12). In other studies, there are also abnormalities or deletions of DNA mismatch repair (MMR) protein, indicating that deficient MMR (dMMR) may explain the hypermutation phenotype observed in tumor samples, which may provide a basis for immune checkpoint inhibitor therapy (8, 13, 14). In addition, mutations in *KMT2C* and *KMT2D* were found in the study, suggesting that the KMT2 histone family may contribute to the appearance of NECC (6, 8, 12). Thus, due to the limited number of samples evaluated to date, there is a genetic complexity and heterogeneity in NECC, with a low overall mutation rate, few copy number alterations, and fewer highly recurrent mutated genes (compared to NEC in other similar histological organs) (15).

Interestingly, the limited NECC genomic data available suggest that mutations cluster in specific gene families and pathways, including the RTK/RAS pathway (*KRAS*, *ERBB2*, *FLT3*, and *ROS1*), the PI3K/AKT/mTOR pathway (*PIK3CA*, *PTEN*, *AKT1*, *AKT2*, and *RICTOR*), the p53 pathway (*TP53*, *ATM*, and *MDM4*), and the MYC pathway (*MYC*, *MYCN*, and *MYCL*) (8). *PIK3CA* encodes the p110α catalytic subunit p110 of phosphatidylinositol 3-kinase (PI3K), and activation of PI3K leads to the production of PIP3 and the further activation of downstream targets (PDK1 and AKT), which can phosphorylate a variety of substrates, including mTOR (16, 17). The PI3K/AKT/mTOR signaling pathway is a recurrent driver pathway that promotes NECC and regulates cell proliferation, differentiation, apoptosis, and carcinogenesis (18–20). Next-generation sequencing has shown that of 19 patients with NECC, approximately 38.78% had at least one mutation in a gene related to the PI3K/AKT pathway, implying that this pathway plays a crucial role in the development of NECC (8). Analysis of the gene set of frequently mutated genes revealed that genes with functions such as small GTPase-mediated signaling, forebrain development, and protein kinase B/AKT signaling were enriched (7). Small GTPase-mediated signaling pathways, such as the RAS/Rho family, play important roles in cellular and developmental processes, including cell proliferation, cytoskeletal dynamics, and angiogenesis, and dysregulation of their transactivation is associated with a variety of cancers (21). Pathways associated with forebrain development may be related to the origin of NECC. which involves neurosecretory cells that perform neuroendocrine integration. Protein kinase B/AKT signaling promotes cell survival and growth in response to extracellular signals that can influence the development and progression of a range of cancers together with mTOR signaling (18, 19). Taken together, the study of the signaling pathway genes mentioned above may contribute to understanding pathogenesis. Nevertheless, given the population-specific nature of somatic mutations in cancer, there remains a dearth of data demonstrating the comprehensive mutational profile at the whole exome level specifically among Chinese patients (22).

Unlike the cervix, the normal endometrium lacks argyrophil cells, and the origin of small cell neuroendocrine carcinoma of the endometrium (NECE) is still unknown (23). Research shows that most NECE are admixed and most of the admixed disease components are non- neuroendocrine carcinoma endometrial cancer (24). For example, Espinosa et al. demonstrated *PTEN*, *KRAS*, *PIK3CA*, *TP53*, and *POLE* mutations in four cases of dedifferentiated endometrial cancer with strong and diffuse neuroendocrine expression (25). Ariura et al. analyzed mutations in only one case of admixed neuroendocrine carcinoma and endometrioid adenocarcinoma, focusing on genetic alterations in mutational hotspots of 50 cancer-related genes and reporting the existence of mutations in *PTEN*, *PIK3CA*, *FGFR3*, and *CTNNB1* (26). Ono et al. analyzed 22 NECE samples and the results of genetic analysis showed several mutations in the NECE group, including *PIK3CA*, *PTEN*, *TP53*, *CTNNB1*, and *KRAS*, which are common in endometrial cancers (27). No significant differences have been identified found in mutations compared to non- neuroendocrine carcinoma endometrial cancers, except for a significant trend in *PIK3CA*, which may suggest that NECE has mutations similar to conventional endometrial cancers rather than pure NEC in other organs (26). Currently, only a few studies have reported the genetic characteristics of NECE and there is still a lack of a genome-wide level assessment.

Both NECC and NECE arise from the diffuse neuroendocrine cell system of female genital tract (28). The aforementioned genomic evidence suggests that, unlike other NECs, NECC and NECE may exhibit similar processes of carcinogenesis, as inferred from their shared dysfunctions involving *PIK3CA*, *PTEN*, *KRAS*, or *TP53*. To date, no formal comparative genomic analysis of NECC and NECE has been reported and the genetic similarity between them is still unclear. In this study, we reported WES data on NECE for the first time and provide an in-depth study of whole exon mutations in 15 Chinese patients of with small cell neuroendocrine carcinoma of different localizations (cervix and endometrium). This study revealed the all-exon characteristics and mutational status of NECC and NECE and identified *PIK3CA* and *KMT2B* as mutations common to tumors of the cervix and endometrium. In addition, a proportion of cervical samples were characterized by abnormal mismatch repair, unlike common cervical cancer, suggesting that abnormal mismatch repair is an important cause of NECC. This study deepens our understanding of the complex molecular composition of NECC and NECE and reveals potential genomic alterations that could be used to better differentiate tumor subgroups and new therapeutic options.

## Results

### Somatic mutation burden and clinical outcomes of NECC and NECE

The detailed clinical information of the 10 patients sequenced for NECC and five NECE samples is in **Table 1**. All of the patients were diagnosed as neuroendocrine carcinoma according to the immunohistochemical staining (IHC) of specific markers, including chromogranin A (CgA), synaptophysin (Syn), CD56, and Thyroid transcription factor-1 (TTF-1) (**Supplementary Figure 1**; **Supplementary Table 1**). All of the NECCs belonged to putative neuroendocrine carcinoma with no other types of neoplastic cells. However, all of the NECEs were accompanied with endometrioid carcinoma component. Above neuroendocrine markers were mostly positive in neuroendocrine carcinoma component (monomorphic neoplastic cells arranged in solid pattern, scant cytoplasm, hyperchromatic and dispersed chromatin, and nuclear moulding and numerous mitoses.) of NECE, while negative in endometrioid carcinoma component (papillary or villoglandular architecture with smooth luminal outline, columnar, abundant cytoplasm, and mild to moderate nuclear atypia.) in the same case. The tumor tissues, of which IHC show neuroendocrine carcinoma component as the major part, were selected for sequencing analysis.

**Table 1:** Clinical characteristics of patients with NECC and NECE.

ID	Age	FIGO stage	Tumor size (cm)	Metastasis	LVSI	HPV^#^	Treatment strategy*	Follow up (m)	Clinical outcome
NECC1	44	IVB	3	Yes (ovarian, pancreatic, mesenteric, omental)	No	High risk	C+S+B+P	23	Survive
NECC3	48	IIIC1	2.5	Yes (LN)	Yes	18+	S+CR+B+P	31	Survive
NECC4	47	IB1	2.3	No	No	18+	S+C+B	55	Survive
NECC5	24	IIIC1	3.3	Yes (LN)	No	18+	S+C	7	Survive
NECC6	27	IB1	1	No	No	16+	S+C	15	Survive
NECC7	51	IVB	6.5	Yes (omental)	No	High risk	S+C+B	34	Died
NECC8	36	IB2	2.5	No	Yes	16+	C+S+B	9	Survive
NECC9	33	IIB	3.5	No	No	High risk	S+CR+B+P	15	Survive
NECC10	39	IB1	1	No	No	18+	S+C	15	Survive
NECC11	49	IVB	8	Yes (LN)	No	18+	CR+B+P	20	Survive
NECE1	63	IVB	3	Yes (pulmonary)	Yes	NA	S+C+R	13	Died
NECE2	56	IIIC2	8	Yes (LN)	No	NA	C+S+P	36	Survive
NECE3	73	IIIB	2	Yes (vaginal)	Yes	NA	S+C+R	16	Died
NECE4	62	IVB	6	Yes (vaginal, vesical, rectal)	No	NA	C+S+R	6	Survive
NECE5	33	IVB	5	Yes (hepatic, pulmonary, peritoneal)	Yes	NA	Palliative care	1	Died

NECC, Small cell cervical neuroendocrine carcinoma; NECE, Small cell endometrial neuroendocrine carcinoma.

FIGO, the International Federation of Gynecology and Obstetrics; LN, Lymph node; LVSI, Lymphovascular space invasion.

* B, Bevacizumab; C, Chemotherapy; CR, Chemoradiation; P, PD-1 inhibitor; R, Radiation; S, Surgery.

^#^ High risk: infected by other 12 high risk type of HPV.

The sequencing depth of tumor specimens and the matched peripheral blood mononuclear cell samples reached 300X (median 298.6, range 199.1–377.9) and 120X (median 120.3, range 74.2–204.6), respectively. In total, we detected 1821 somatic mutations in NECC patients and 3550 in NECE patients. The median tumor mutation burden (TMB) of NECC was 1.3, which is comparable to a previous report (12). One of 10 NECC samples and three of five NECE samples showed hypermutation phenotype (> 10 mut/Mb). We further verified whether there were germline or somatic mutations in DNA mismatch repair genes in these hypermutated samples, and found one somatic *MSH6* frameshift deletion in one hypermutated NECE sample (NM_001281492: c.2864delC) and a somatic *MSH2* frameshift insertion in the hypermutated NECC sample (NM_000251: c.594_595insGCTGACATATCAT).

Among the remaining nine non-hypermutated NECC samples, we found that the total number of somatic mutations was significantly higher in patients diagnosed with the International Federation of Gynecology and Obstetrics (FIGO) stage III–IV (average 188) compared to stage I–II (average 47.25, **Supplementary Table 1**) (*p* = 0.047, t-test). No relationship was found between the number of NECC mutations with age of diagnosis, metastasis, or Ki-67 index, although patients with metastasis showed trends of higher mutation burden (*p* = 0.077, t-test). Among the NECE samples, the patient with the lowest burden of tumor mutation exhibited the highest Ki-67 index (**Supplementary Table 1**).

### Mutational signature of NECC and NECE in Chinese patients

**Figure 1:**
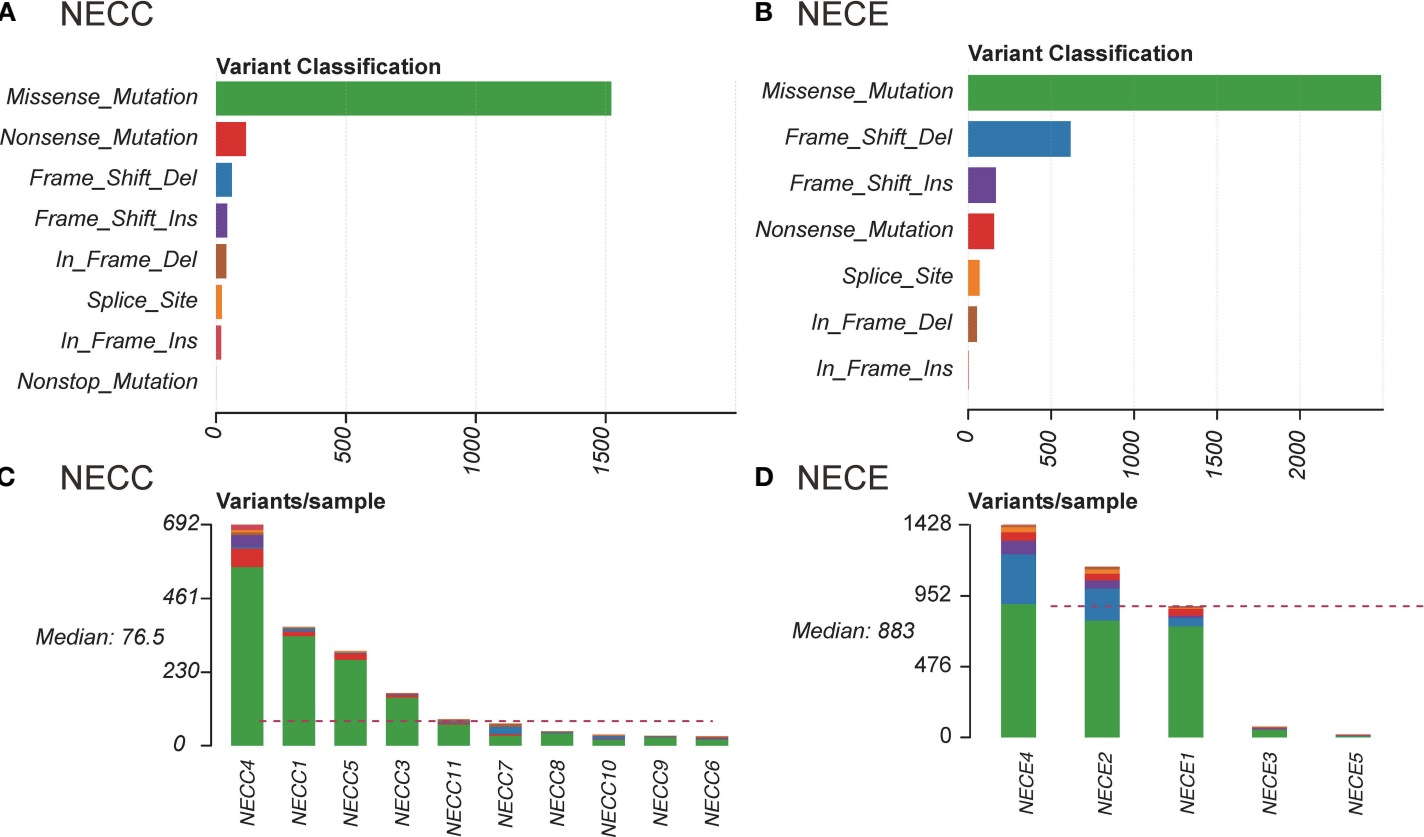
Distribution of somatic mutations in NECC and NECE. **(A)** Functional influence of somatic mutations in NECC, the X-axis indicates the number of mutations belonging to each of the mutation type (Y-axis). **(B)** Functional influence of somatic mutations in NECE. **(C)** Distribution of somatic mutations in each sample of NECC. The color code is the same as that of **(A)**. The X-axis indicates the number of somatic mutations in each patient. **(D)** Distribution of somatic mutations in each sample of NECE. The color code is the same as that of **(B)**.

We also examined the proportion of each somatic mutation annotation type in the exon regions of patients with NECC and NECE (**Figure 1**). Missense mutations were the most abundant type in both NECC and NECE. In NECC samples, nonsense mutations were the second most abundant, whereas frame shift deletions were the second most frequent in NECE (**Figures 1A, B**). As shown in **Figure 1D**, most frame shift deletions were detected in the three hypermutated NECE samples, suggesting a high microsatellite instability (MSI-H) status in these tumors (29). Furthermore, in the hypermutated NECC sample, we observed a high proportion of frame shift insertions (**Figure 1C**).

**Figure 2:**
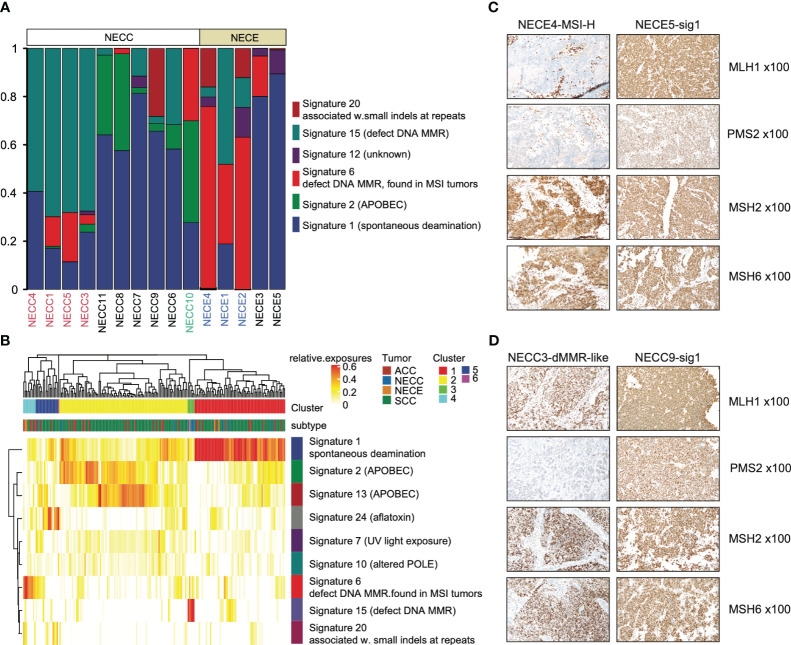
Mutational signature distribution across patients and tumor types. **(A)** Mutational signature burden of NECC and NECE. The X-axis indicates each patient with white as NECC and grey as NECE. Red font shows the mismatch repair deficiency type of NECC and blue font indicates micro-satellite instability type of NECE. The Y-axis indicates accumulated proportion of each mutational signature shown in the color legend. **(B)** Unsupervised clustering of patients according to mutational signature burden. Color legend in X-axis shows tumor types and cluster results. Color legend in Y-axis showed mutational signatures. **(C)** Immunohistochemical staining images (x100) of two subtypes of NECEs. Sample ID and its subtype is labeled on the top. MSH1 and PMS2 shows negative staining in MSI-H sample. **(D)** Immunohistochemical staining images (x100) of two subtypes of NECCs. Sample ID and its subtype is labeled on the top. Despite PMS2 displaying relatively weak staining in dMMR-like samples, it should still be deemed positively stained according to the distribution of the positive cells.

For single nucleotide variations (SNV), C > T represents 76% and 62% of total NECC and NECE mutations, respectively. We then performed mutational signature deconvolution by non-negative matrix factorization to identify the mutational processes for SNV in these two tumors. As shown in **Figure 2A**, six COSMIC mutational signatures were factorized from a total of 15 samples of these two tumors (signature-specific cutoff = 0.008). NECC and NECE can be divided into three or two types according to loads of the mutational signatures, respectively. For NECC, the COSMIC signature 15, which is attributed to the DNA dMMR, contributed as the major process in four patients. The remaining six patients contains five with spontaneous deamination (COSMIC signature 1) and one with APOBEC (COSMIC signature 2) as major mutational signature. Consistent with the estimated dMMR by mutational signature loading, the four patients harbored significantly more mutations compared to the other six patients (mean 381.75 vs 49, *p* = 0.005, t-test) (**Supplementary Table 1**). For NECE, the three hypermutated and MSI-H patients also showed a high proportion of COSMIC signature 6 (dMMR found in MSI tumors), while the remaining two NECE samples exhibited signature 1 as the major type.

A previous report showed that NECC shared a similar mutational process with other types of cervix cancer, including endocervical adenocarcinoma (ACC) and squamous carcinoma of the cervix (SCC) (12). We also examined the similarity in our samples. As shown in **Figure 2B**, both NECC and NECE samples with signature 1 as the major type clustered with ACC and SCC. Moreover, two distinct clusters were formed by dMMR NECC (cluster 3) and MSI-H NECE (cluster 4) samples, respectively. This observation indicated that there are significantly different carcinogenesis processes occur within NECC and NECE. In patients with available samples (9/10 NECCs and 3/5 NECEs), we conducted IHC of four MMR key genes, including MLH1, PMS2, MSH2, and MSH6, and noticed an exact staining-signature correlation in NECE. Two available MSI-H patients demonstrated a lack of MLH1 and PMS2, while the remained signature 1 type did not (**Figure 2C**; **Supplementary Table 1**). However, we did not find such correlation in NECCs. Although it appears that the dMMR NECCs exhibited weak staining of PMS2, this type should still be the identified as positively stained according to standard clinical diagnostic procedures (**Figure 2D**; **Supplementary Table 1**). Consequently, we divided NECC samples into two subtypes, including dMMR-like type and signature 1 type. NECC10, the only one patient with APOBEC as the primary signature, was also assigned to signature 1 type, as signature 1 becomes the major signature under a more stringent signature-specific cutoff of 0.03 (**Supplementary Figure 2**; **Supplementary Table 2**).

We further analyzed the potential clinical significance of NECC subtypes. The dMMR-like subtype exhibited more pronounced metastasis (3 out of 4 cases) and elevated FIGO stages (III or IV, 3 out of 4 cases) in comparison to the signature 1 subtype (metastasis in 2 out of 6 cases, FIGO stages III or IV in 2 out of 6 cases). Nevertheless, these more severe manifestations did not attain statistical significance (*p* = 0.26, Fisher’s exact test), a circumstance that may be attributed to the constraints of our sample size. Furthermore, there existed no substantial variance in tumor size at the time of diagnosis between these two subtypes (*p* = 0.83, Wilcox-rank test). These observations hint at the possibility of the dMMR-like subtype manifesting more severe clinical presentations, a hypothesis that necessitates validation in larger cohorts.

Previous studies on NECC of European ancestry have indicated that the mutational signatures related to the activation of APOBEC as the primary type (12, 15). However, in our cohort, we have only observed limited contribution of APOBEC. In the dMMR-like type, APOBEC had a contribution of less than 1% in three out of four patients, and the highest contribution is nearly 3% (**Figure 2A**; **Supplementary Table 2**). In signature 1 type, three out of six patients showed APOBEC contribution of less than 10% (**Figure 2A**; **Supplementary Table 2**). Since all 10 NECC patients in our analysis were diagnosed with high-risk human papillomavirus (HPV) infection, the above findings indicated that the anti-HPV infection process of APOBEC had limited contribution to the carcinogenesis of NECCs in our cohort, especially for the dMMR-like type (30).

### HPV integration and aneuploidy

**Figure 3:**
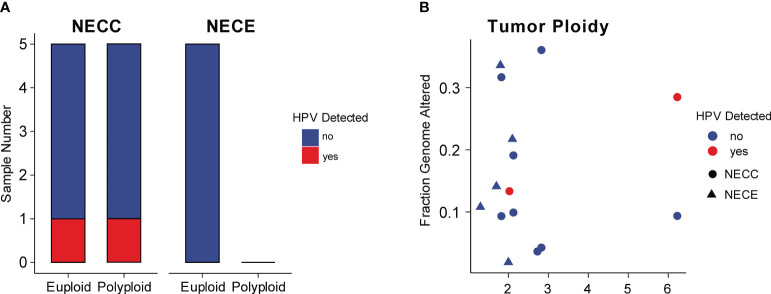
HPV integration and aneuploidy of NECC and NECE. **(A)** Number of patients (X-axis) with euploidy, polyploidy, and HPV integration in NECC and NECE specimens. **(B)** Tumor ploidy (X-axis) and the fraction of genome with copy number alterations (Y-axis). The circle indicates NECC and the triangle indicates NECE. Colors show tumors with (red) or without (blue) HPV integration.

Integration of HPV is a critical factor in cervical carcinogenesis (31). We checked the HPV integration events in our NECC samples. Although high-risk HPV infection was detected in all of our patients and seven were identified HPV16 or HPV18 (**Supplementary Table 1**), the HPV-human genome integration events were only detected in two patients, both of whom belonged to signature 1 type and infected by HPV16 (**Figure 3A**; **Supplementary Table 1**). Compared to previous reports, the integration event in our data was lower (20% vs. 53%) (12). The integration sites were annotated in *NR4A2* and *PGAP3* (**Supplementary Table 1**). The up-regulation related to HPV integration of *NR4A2* has been reported as a common event of SCC, which indicated the critical role of *NR4A2* in the oncogenesis of NECC (32, 33).

High-risk HPV infection caused aneuploidy and viral integration in the cancer genome was believed to be a major carcinogenesis process of SCC (34). As reported elsewhere, NECC also showed a significant association between aneuploidy and viral integration (12). However, in our data, we did not observe such linkage, since among our cases only one out five polyploidy and one out of five euploidy NECC had HPV integration, respectively (**Figure 3A**). The patient with HPV integration sites annotated in *NR4A2* showed the largest polyploidy status. Furthermore, all NECE tumors were euploidy and no type of dMMR-like of NECC had polyploidy greater than three, which is consistent with the observation of an inverse association between MSI and aneuploidy in various cancers (**Figure 3B**) (35). Together with the analysis of the mutational signature, these findings indicated that dMMR is a major carcinogenesis factor in part of NECC from Chinese patients.

### Somatic copy number alterations and structural variations

We further analyzed somatic copy number alterations (SCNAs) in NECC and NECE using GISTIC2.0, respectively (36). Due to the limited sample size, neither of these two carcinomas had focal SCNA identified with q < 0.05. There were three recurrent SCNAs detected in NECC (*q* < 0.15, altered in no less than 40% patients, **Supplementary Figure 3**), including deletions of 6p21.32 (*HLA-DRB5*), 11q25, and 15q21.2 (*TRPM7*). For NECE, only two recurrent deletions were found (*q* < 0.15, altered in no less than 40% patients, **Supplementary Figure 3**), including 1q36.32 (*TNFRSF14*) and 11p15.4 (*WEE1*). These SCNAs may lead to the impairment of immune response (*HLA-DRB5* and *TNFRSF14*) and cell cycle control (*TRPM7* and *WEE1*) in NECC and NECE.

Upon further classification of NECCs into the aforementioned two subtypes, it was observed that in signature 1 type, there were no focal SCNAs identified with a significance threshold of *q* < 0.25. This suggests the SCNAs may serve as passenger events in this particular subtype. Conversely, in the dMMR-like subtype, we detected eight focal somatic deletions (*q* < 0.15, altered in no less than 50% patients, **Supplementary Figure 3**). In addition to 6p21.32 (*HLA-DRB5*) and 15q21.2 (*TRPM7*), other loci included 1p36.33, 2p13.1 (*DCTN1*), 3p25.3 (*FANCD2*), 3q12.3, 3q13.33 (*POLQ*), and 7q22.3. The deletions of *FANCD2* and *POLQ* further support the dysfunction of DNA repair system of this subtype.

Somatic structural variations (SV) were called by SvABA (37). Among the total of 15 patients, we only detected four SVs in the four dMMR-like NECC patients under the criterion described in MATERIALS AND METHODS (**Supplementary Table 3**). No SV was detected in NECEs possibly due to the absence of breakpoints within exome regions. The four SVs included duplication of 15:44,801,470–44,881,820 in NECC1, which may fuse *CTDSPL2* (NM_016396.3) and *SPG11* (NM_025137.4); homology recombination of *CBL* (NM_005188.4) in NECC3, leading to a potential fusion of CBL exon 8 with its own exon 10 in a reverse manner; deletion of *MUC17* (NM_00104015.2) exon 3 in NECC4; and deletion of *TREH* (NM_007180.3) intron 4 (**Supplementary Figure 4**). It is noteworthy that *CBL* is a proto-oncogene known to impact JAK2, EGFR, and PI3K signaling pathways (38). *CTDSPL2* has been reported as tumor suppressor, involved in restraining tumor growth in pancreatic cancer (39). *MUC17*, one of the 21 mucin genes, also exhibits tumor suppressor properties (40). These findings provide further evidence supporting the contribution of SVs to the carcinogenesis of the dMMR-like subtype of NECCs.

### Recurrent mutated genes in NECC and NECE showed similar carcinogenesis processes

**Figure 4:**
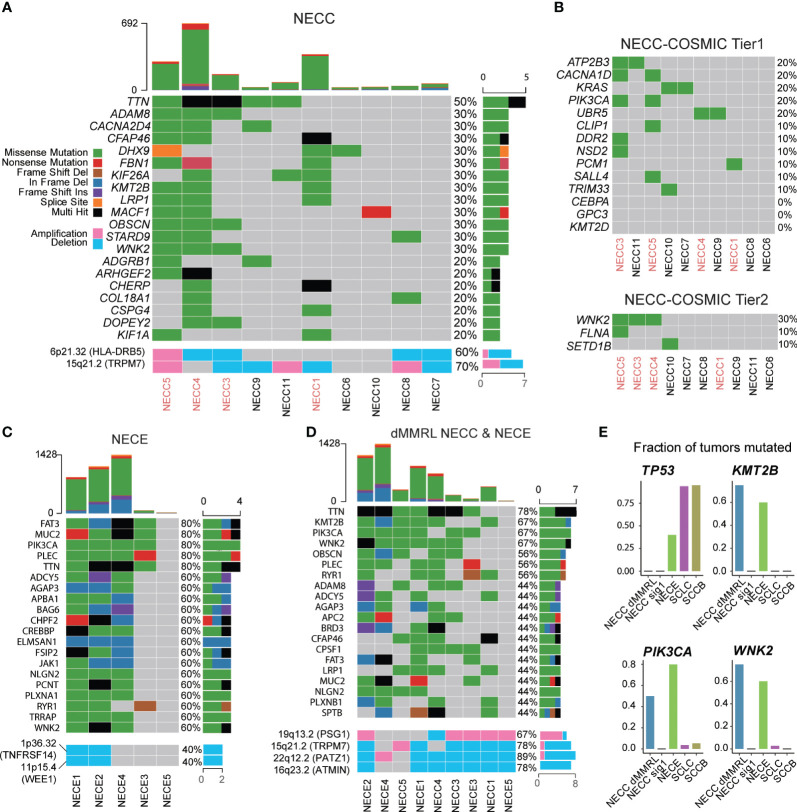
Somatic mutational landscape of NECC and NECE. **(A)** The top 20 genes with high somatic mutation burden and focal SCNAs in NECC. The colors indicate different functional catalogue of mutations as shown in the colored legend. The percentage of mutated tumors are on the right. The red font of the X-axis indicates mismatch repair deficiency type of NECC. **(B)** Somatic mutation burden among COSMIC Cancer Gene Census Tier 1 and 2 in NECC. **(C)** The top 20 genes with high mutational burden and focal SCNAs in NECE. **(D)** The top 20 genes with high mutational burden and focal SCNAs in mismatch repair deficiency-like subtype of NECC and NECE. **(E)** Fraction of mutated samples in different tumor types for recurrent mutated genes of mismatch repair deficiency-like NECC and NECE. dMMRL: mismatch repair deficiency-like.

Next, we analyzed the recurrent mutated genes in NECC and NECE, respectively, to identify possible molecular processes involved in the carcinogenesis of these two tumors. For NECC, with the exception of *TTN*, which has the largest coding region among all human genes and was mutated in 50% of patients with NECC, no mutated genes contributed to more than 40% of the patients (**Figure 4A**). There were 12 genes mutated in 30% of patients, of which only *WNK2* belonged to the COSMIC Cancer Gene Census (Tier 1 or Tier 2, **Figures 4A, B**). When we limited the analysis to the genes of the COSMIC Cancer Gene Census Tier 1, only four genes, including *ATP2B3*, *CACNA1D*, *KRAS*, and *PIK3CA*, showed recurrent mutations only in two patients (**Figure 4B**). No *TP53* mutations were found in the 10 patients. This was consistent with the immunohistochemical observation that no mutated P53 staining in the tumor tissues was detected in patients with available results (**Supplementary Table 1**). Moreover, among the aforementioned genes displaying SCNAs, namely *HLA-DRB5* and *TRPM7*, deletions were observed in 40% of patients, while amplifications were detected in 10% and 30% of patients, respectively. (**Figure 4A**). These observations were consistent with previous reports that recurrent mutated genes were limited and only contributed to a small proportion of patients (< 30%) (8, 12).

We further examined recurrent mutations in different subgroups of NECC. At the COSMIC Cancer Gene Census gene level, *CACNA1D*, *PIK3CA*, and *WNK2* mutations belonged to the dMMR-like type of NECC. RAS mutations only occurred in signature 1 type NECC, which affected 67% of the patients (4/6) including two with *KRAS* mutations, one with the *NRAS* mutation, and one with the *HRAS* mutation (**Supplementary Figure 5**). At the whole gene level, *WNK2*, *KMT2B*, *TTN*, *ADAM8*, and *LRP1* were mutated in three of four dMMR-like NECC patients. No gene was mutated in more than two patients with the signature 1 type. The above observations, especially the fact that *PIK3CA* and RAS mutations appeared exclusively in different subtypes of NECC, further indicated the different molecular mechanism of carcinogenesis of these two subtypes of NECC.

For NECE, five genes were mutated in 80% of patients, including *PIK3CA*, *FAT3*, *MUC2*, *PLEC*, and *TTN*, among which *PIK3CA* and *FAT3* belong to the COSMIC Cancer Gene Census gene tier 1 and 2, respectively (**Figure 4C**). All patients with MSI-H harbored mutations of *PIK3CA*, *BRD3*, *CREBBP*, *CTCF*, *JAK1*, *TRRAP*, *FAT3*, and *WNK2* (**Figure 4C**). This observation indicated that the PI3K/AKT signaling pathway (*PIK3CA* and *JAK1*) and the JAK/STAT signaling pathway (*PIK3CA*, *JAK1* and *CREBBP*) may play an important role in the carcinogenesis of the MSI-H type of NECE. The genes related to chromatin remodeling activity, including *KMT2A*, *KMT2D*, and *SETD2*, were mutated only in two of three MSI-H type of NECE. Additionally, the recurrent deletion of *TNFRSF14* and *WEE1* were also observed only in two of three MSI-H type of NECE (**Figure 4C**). Furthermore, *PIK3CA* and *PTEN* mutations, which are critical genes in the PI3K/ATK signaling pathway, were also found in the signature 1 type of NECE. This result further suggested that PI3K/AKT dysfunction may contributed to the formation of all types of NECE tumors, which is consistent with previous reports (25–27). There were two patients with *TP53* mutations, one of which belonged to the MSI-H type and one of the signature 1 type (**Figure 4C**). The estimated mutation rate for *TP53* (40%) is comparable to previous results obtained from candidate gene sequencing from 22 NECE patients (27). The fact that both the MSI-H type and the signature 1 type had *TP53* mutation also indicates that the dysfunction of *TP53* signaling is a common causal factor of NECE tumors.

We further combined the dMMR-like type of NECC and NECE to determine whether there was a commonality in tumorigenesis between these two cancers. As shown in **Figure 4D**, except for *TTN*, *KMT2B* and *WNK2* were mutated in three of four dMMR-like NECC and in three of five NECE samples. Furthermore, *PIK3CA* was mutated in two of four dMMR-like NECC and in four of five NECE samples. This observation indicated the important role of these three genes and their related molecular functions in NECC and NECE. *PIK3CA* is the key factor in the PI3K/AKT signaling pathway; previous reports have shown that mutations in this gene account for ~15%–30% of patients with NECC and 50% of patients with NECE (8, 10, 12, 27). *WNK2* encodes a cytoplasmic kinase involved in various carcinogenesis processes, including cell cycle progression, antiapoptotic mechanisms, invasion, and metastasis (41). *KMT2B* is an important methyltransferase gene that contributes to chromatin remodeling dysfunction in various cancers (42). We further compared the frequency of mutations of these three genes in NECC, NECE, and public data of neuroendocrine tumors of the lung (SCLC) and bladder (SCCB). As shown in **Figure 4E**, the mutation frequencies of these three genes are significantly low in SCLC and SCCB compared to dMMR-like type of NECC and NECE (43, 44). Moreover, through our analysis, we detected five significant focal SCNAs (*q* < 0.05, **Figure 4D**; **Supplementary Figure 3**): amplification of 19q13.2 (*PSG1*) and deletions of 15p21.2 (*TRPM7*), 16q23.2 (*ATMIN*), 19p13.2, and 22q12.2 (*PATZ1*). This observation indicated the potential importance of *PATZ1*, which is known to be involved in chromatin remodeling and can interact with *TP53* to regulate proliferation and DNA damage response (45). Our observation indicated that the dysfunction of the PI3K/AKT pathway, enhanced cell cycle and antiapoptotic activity, and abnormal chromatin remodeling were the major common mechanisms of the dMMR-like type of NECC and NECE.

## Discussion

In this study, we provide a mutational landscape of NECC and NECE in Chinese patients. TMB is a predictive marker for various clinical outcomes of solid tumors (46). In ovarian cancer, a higher TMB is associated with lower FIGO stages, which may lead to an increase in immune infiltration in such patients (47). However, in NECC, we found that the higher TMB is associated with higher FIGO stages. This phenomenon was primarily attributable to the dMMR-like subtype of NECC, which exhibited elevated TMB and a more severe metastatic profile (**Supplementary Table 1**). The four patients classified as dMMR-like subtype also ranked as the top four with the highest TMB levels. Furthermore, although it did not reach statistical significance, the dMMR-like subtype displayed a tendency toward increased metastasis compared to the signature 1 type (3 out of 4 cases vs. 2 out of 6 cases, *p* = 0.26, Fisher’s exact test). Metastasis plays a pivotal role in determining FIGO stage. Thus, the dMMR-like subtype may be linked to more adverse clinical outcomes.

As shown in **Supplementary Table 1**, seven out of 15 of our WES data were generated from formalin-fixed paraffin-embedded (FFPE) tissues. It is well accepted that FFPE process will induce artificial C > T substitutions in sequencing data (48). In our data, after induce reparation process during sequencing library preparation (MATERIALS AND METHODS), we did not observe the increase in the number of somatic mutations in FFPE samples within each tumor type (NECC, *p* = 0.265, t-test; NECE, FFPE median 447.5 vs. fresh frozen 883). A recent report shows that unrepaired and repaired FFPE library will exhibit an excess of COSMIC signature 30 and 1, respectively (49). The absence of signature 30 in our work further indicated a successful repair process (**Figure 2A**). Additionally, the excess of signature 1 did not affect the subgrouping of our samples, especially for dMMR-like NECCs, because four out of seven of the FFPE data did not show signature 1 as the primary type and the only one FFPE NECC with signature 1 as the primary type showed signature 20, which is not related to DNA mismatch repair, as the second abundant (**Figure 2A**; **Supplementary Table 1**).

The relationship between HPV and NECC remains to be elucidated. High-risk HPV has been reported to be associated with most cervical cancers, including SCC and ACC. Recent studies have found that NECC is associated with high-risk HPV, mainly HPV16 and HPV18 types (50). Although HPV18 infection was found to be statistically more common in patients with NECC than in patients with other histological subtypes, no significant differences were found in the mutational profiles of NECC with and without HPV18 (11). In our data, 10 NECC patients were diagnosed with high-risk HPV infection, of which five were infected with HPV18 and two with HPV16. This observation is consistent with previous reports. Previous WES analysis found a higher load of APOBEC-related mutational signatures and a statistically significant association between detectable HPV integration and tumor cell aneuploidy, suggesting that in NECC HPV infection may play a role in disrupting genomic integrity and promoting tumor formation (12). Another study sequenced 50 cancer genes in a larger sample size and found that NECC showed a different mutation pattern with other cancers caused by HPV infection. The authors hypothesized that HPV in NECC may simply reflect carrier status rather than being a causal factor (9). In our study, we found that only two patients had HPV integration events by WES, which is less than that reported in previous WES reports. Moreover, APOBEC related signature showed limited contribution (relative exposure < 0.1) to majority patients (7/10) in a lenient signature specific cutoff of 0.008 (**Figure 2A**; **Supplementary Table 2**) and even absent under a stringent cutoff of 0.03 (**Supplementary Figure 2**; **Supplementary Table 2**). In addition, aneuploidy was related to dMMR rather than HPV integration. Previous reports showed that *PIK3CA* mutations play a critical role in HPV-induced carcinogenesis in SCC, ACC and head and neck cancers (32, 51). However, in our data, *PIK3CA* mutations appeared only in the dMMR-like type of NECC (4/10) and none were observed in the signature 1 type of NECC (6/10). These observations suggested that HPV infection is not the causative factor of NECC, at least in Chinese patients. Furthermore, genomic integration events were present in both of the patients infected with HPV16 (**Supplementary Table 1**). In addition, one of the integrated genes, *NR4A2*, has also been reported as the integration hotspot in SCC (32, 33). Consequently, further analysis is needed to determine whether HPV integration is critical for HPV16 infection-related NECC.

Taking advantage of WES, we constructed a mutational signature landscape of NECC and NECE that corresponds to the entire coding region in Chinese patients. We divided both NECC and NECE into two subtypes (**Figure 2B**). Previous research has identified dMMR mutations in several patients with NECC, but the mutational profile of these patients has not been described (12, 52). In our study, for the first time, we noticed that NECC patients can be grouped into dMMR-like type according to their mutational signature loading status. Further, the dMMR-like type showed a significantly different mutational pattern compared to the signature 1 type. Although harboring more mutations, the RAS gene family mutations is absent in the dMMR-like type of NECC. Four of the six signature 1 types of NECC patients had RAS mutations, including two in *KRAS*, one in *NRAS*, and one in *HRAS*. Furthermore, the above-mentioned *PIK3CA*, *WNK2*, and *KMT2B* mutations can only be seen in the dMMR-like type of NECC. The dysfunction of PI3K/AKT signaling and RTK/RAS signaling is the main cause of NECC (8). Data describing mutational profiles also confirmed that *PIK3CA* and RAS mutations appeared exclusively in different patients (8, 12). Our work further suggested that PI3K/AKT contributed to the type of dMMR-like and RTK/RAS contributed to elucidate the type of signature 1, respectively, which could explain the exclusivity of its appearance. Furthermore, these observations also suggested that molecular subtyping is critical in the treatment of NECC, as mutations in genes of the *PIK3CA* and RAS families are distinct therapeutic targets (8).

NECE patients can also be divided according to the MSI-H type and the signature 1 type. However, the signature 1 type only included two patients with one showing an extremely low mutation burden; thus, we could not obtain a reliable mutational profile of the type signature 1 type. All types of MSI-H in NECE patients harbored mutations in the PI3K/AKT and JAK/STAT signaling pathways and majority (2/3) had mutations involving chromatin remodeling genes. Although the functional relationship between PI3K/ATK signaling and MMR is not clear, enriched *PIK3CA* mutations were also observed in tumors with dMMR in colorectal cancer (53). Due to its low prevalence, genomic studies focusing on NECE are still scarce. Our work, although only five patients were included, may provide a clue about the mutational landscape of NECE in coding regions. We found that PI3K/AKT mutations and TP53 mutations may contribute to the formation of NECE regardless of subtypes, and JAK/STAT and chromatin remodeling dysfunction may only be related to the MSI-H type of NECE.

Discordant with previous research, in our NECC patients, we did not identify mutations in *TP53* or *KMT2D* (8, 10, 12, 15, 52). The frequency of *TP53* mutation varies from ~8%–40% according to different reports. Research based on Chinese patients containing the largest sample size (22 patients) showed that the *TP53* mutation exists nearly exclusively with the *KRAS* (none sharing patient) and *PIK3CA* (only one shared patient) mutations (8). These results suggest that patients with the *TP53* mutation may belong to another subtype of NECC, which was not present in our samples. Furthermore, the recurrent deletion of *PATZ1*, an interacting gene of *TP53*, in both the dMMR-like subtype of NECC and NECE, highlights the potential significance of TP53-related pathway dysfunction as a prominent event in the carcinogenesis of these two tumors. Although no *KMT2D* mutations were detected, we found a high frequency of *KMT2B* mutations in the dMMR-like subtype of NECC. Although they form different protein complexes and binding to different regulatory regions of genes, both *KMT2B* and *KMT2D* are critical components related to chromatin remodeling activity (42). Mutations in *KMT2B* in our samples can also indicate the dysfunction of this activity, especially in the dMMR-like type of NECC.

By comparing recurrent mutated genes in NECC and NECE, we found that mutations in *PIK3CA*, *KMT2B*, and *WNK2* were of high frequency in both the dMMR-like type of NECC and NECE (**Figure 4D**). This observation indicates that these two gynecological neuroendocrine tumors may share a common carcinogenesis process, including PI3K/AKT signaling dysfunction, abnormal chromatin remodeling activity, and enhanced cell cycle and antiapoptotic functions. Among these genes, *PIK3CA* has been repeatedly reported in both NECC and NECE. *KMT2B* and *WNK2* are newly identified by our analysis. To further investigate whether mutations in these three genes are common in neuroendocrine tumors, we compared their mutation frequencies in NECC, NECE, SCLC, and SCCB. The substantially lower mutation frequencies in SCLC and SCCB indicate that these gene mutations are likely to be causal events specifically in gynecological neuroendocrine carcinomas (**Figure 4E**).

A major limitation of our research is the relatively small sample size. The restricted sample size prevented us from establishing precise associations between the NECC subtypes and their clinical characteristics. Specifically, given the limited cohort and uncertain prognosis, we were unable to conduct analyses on disease-free survival and overall survival (**Table 1**; **Supplementary Table 1**). However, our work can still provide valuable information on the mutation landscape, especially molecular subtyping, of these two rare tumors. Future work enrolling larger samples is urgently needed for a more detailed analysis of the relationship among mutations, carcinogenesis of these two tumors, and their clinical outcomes. Moreover, another limitation is lacking direct functional evidence of dMMR-like type of NECC. Unlike MSI-H NECE, dMMR-like NECC did not show negative staining of the four key MMR genes in IHC (**Figures 2C, D**). MMR signaling pathway is complex system, future work focusing on its changes in dMMR-like NECC is critical for molecular subtyping of this rare tumor.

In conclusion, we analyzed the mutational landscape in coding regions of NECC and NECE and stratified these tumors into different subtypes according to their mutational signatures. Recurrent analysis of mutated genes identified gene mutations in PI3K/AKT signaling, cell cycle and antiapoptotic processes, and chromatin remodeling activity, which suggests these are shared carcinogenesis processes in both NECC and NECE.

## Materials and methods

### Sample collection and ethics statement

In this study patients with primary NECC or NECE were recruited. Tissue samples used in this work were collected from the Peking Union Medical College Hospital. The patients were diagnosed as NECC or NECE according to hematoxylin and eosin staining and IHC of CgA, Syn, CD56, and TTF-1. Histopathological diagnosis and tumor cell content were independently reviewed by two pathologists. For all patients, tumor samples adjacent to slides with an estimated tumor content greater than 50%, and for NECEs, tumor tissues adjacent to IHC slides showing majority of tumor cells were neuroendocrine carcinoma, were used for WES.

This study was conducted in accordance with the principles of the Declaration of Helsinki and was approved by the ethics committees of the Peking Union Medical College Hospital. All patients gave their informed consent in writing and the ethics committees approved the consent procedure.

### DNA extraction and whole exome sequencing

Whole blood samples from recruited patients were collected using an ethylenediaminetetraacetic acid blood collection tube (BD, Franklin Lakes, NJ) and stored at -80°C before sequencing. Among the tumor tissues collected, eight (seven NECC and one NECE) were stored in liquid nitrogen immediately after the surgical procedure before sequencing, and seven (three NECC and four NECE) were FFPE tissues (**Supplementary Table 1**). DNA from blood and tumor tissues was extracted using the QIAamp DNA Mini Kit (Qiagen, Hilden, Germany) according to the manufacturer’s protocol. The DNA from FFPE tissues underwent a repair process using NEBNext FFPE DNA Repair Mix (New England Biolabs, Ipswich, MA). The concentration and quality of the extracted DNA were measured using the Agilent 2100 assay (Agilent, Santa Clara, CA).

WES libraries were built using the Agilent SureSelect human all-exome V6 kit (Agilent, Santa Clara, CA) according to the protocols. Subsequently, target-enriched sequencing libraries were sequenced on NovaSeq 6000 (Illumina, San Diego, CA) with 150-bp paired-end protocols. The estimated target coverage was 250X for tumor samples and 100X for matched whole blood samples.

### Sequencing data analysis

Sequencing reads in FASTQ format were subjected to a quality control process using fastQC (v0.11.09) and Cutadpt (v4.2) (54). Adapter sequences and low Q-score bases (Q < 20) in each read and low-quality read pairs (any read with remaining base less than 70 bp) were removed accordingly. The trimmed reads were aligned with human reference genome build GRCh37 plus HPV16 or HPV18 sequences with the BWA algorithm (v0.7.11) (55, 56). Somatic mutations were called by GATK mutect2 using default parameters (57). The somatic mutations obtained, which were absent in dbSNP149, were used for further analysis. Somatic mutations of ACC, SCC, SCLC, and SCCB were obtained from their original publications (43, 44, 58).

Mutational signatures were derived from somatic single nucleotide variants and their adjacent bases using non-negative matrix factorization (59). Only coding variants were used for this analysis. The YAPSA R package was used to estimate the relative contribution of each COSMIC mutational signature to all samples in this work. Two signature-specific cutoffs of YAPSA were selected, including 0.008 (lenient) and 0.03 (stringent).

MACS2 was used to identify reads aligned with HPV genomes and pinpoint tumor genomes with HPV integration (60). The integration sites were annotated according to chimeric reads as described elsewhere (61). Only integration sites which were supported by at least three non-redundancy reads were defined as true integration events. The polyploidy status of tumor samples was estimated by Sequenza (v2.1) (62).

The copy number statues of each tumor were determined using CNVkit (63). Subsequently, the assessment of focal SCNAs was conducted using GISTIC 2.0, employing copy number gain and loss thresholds of 0.3 and a confidence level of 0.90 (36). The focal SCNAs in the combined dMMR-like NECC and NECE were defined as q < 0.05 and recurrent altered in at least two dMMR-like NECCs and two NECEs. For the analysis of SVs, SvABA was employed, utilizing a targeted strategy for somatic SV detection (37). In addition to the default filtration criterion, SVs meeting the following conditions were excluded from further analysis: breakpoints supported by fewer than 10 reads, breakpoints located within repeat regions, or SVs only detected through discordant read pairs.

### Immunohistochemical staining

FFPE tissue slides of NECC and NECE were subjected to IHC to diagnose neuroendocrine carcinomas and dMMR. Four markers, including CgA (ZM-0076; ZSGB-BIO, Beijing, China), TTF-1 (MAB-0599; MXB, Fujian, China), CD56 (PA0191; Leica Biosystem, Heidelberger, Germany), and Syn (PA0299; Leica Biosystem, Heidelberger, Germany), were employed to diagnose neuroendocrine carcinomas. In order to diagnose dMMR, four markers, MLH1 (ZM-0152; ZSGB-BIO, Beijing, China), PMS2 (ZM-0407; ZSGB-BIO, Beijing, China), MSH6 (ZA-0541; ZSGB-BIO, Beijing, China), and MSH2 (ZA-0702; ZSGB-BIO, Beijing, China), were utilized. The stained slides were evaluated by two experienced pathologists, who were unaware of the identities of the samples, independently.

### Statistical analysis

All statistical analyses were performed with R 4.0. Statistical significance was defined as *p* < 0.05.

## Data availability statement

WES data generated for this study have been deposited in the Genome Sequence Achieve at the National Genomics Data Center with accession number PRJCA015298, which is publicly accessible at https://ngdc.cncb.ac.cn/bioproject/.

## Ethics statement

The studies involving humans were approved by the ethics committees of the Peking Union Medical College Hospital. The studies were conducted in accordance with the local legislation and institutional requirements. The participants provided their written informed consent to participate in this study.

## Author contributions

WW, WC, YJ, and LP contributed to conception and design of the study. YL, BC, YG, YS, and YPL contributed to the diagnosis of patients, collection of the samples, and the generation of the sequencing data. FZ and WC performed the bioinformatics analysis. WW and FZ wrote the manuscript. All authors contributed to the article and approved the submitted version.
