# Supplementary material for: Whole exome sequencing identifies common mutational landscape of cervix and endometrium small cell neuroendocrine carcinoma
Source: Front Oncol. 2023 Oct 18;13:1182029. doi: 10.3389/fonc.2023.1182029 (PMC10618670; doi:10.3389/fonc.2023.1182029)
Supplement: Supplementary file 1 [file DataSheet_1.docx]

Supplementary Figure 1. Immunohistochemical staining images (x100) of four markers of neuroendocrine carcinomas. Both tissues showed positive staining for all four markers except CD56 of NECC11.


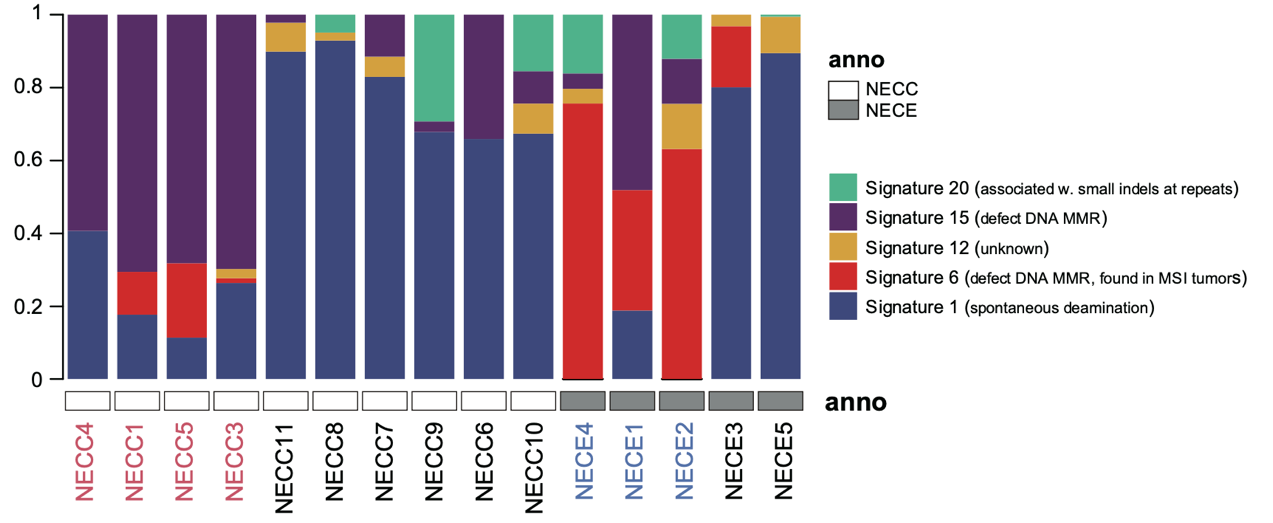


Supplementary Figure 2. Mutational signature burden of NECC and NECE under the signature-specific cutoff of 0.03. The X-axis indicates each patient with white as NECC and grey as NECE. Red font shows the mismatch repair deficiency type of NECC and blue font indicates micro-satellite instability type of NECE. The Y-axis indicates accumulated proportion of each mutational signature shown in the color legend.


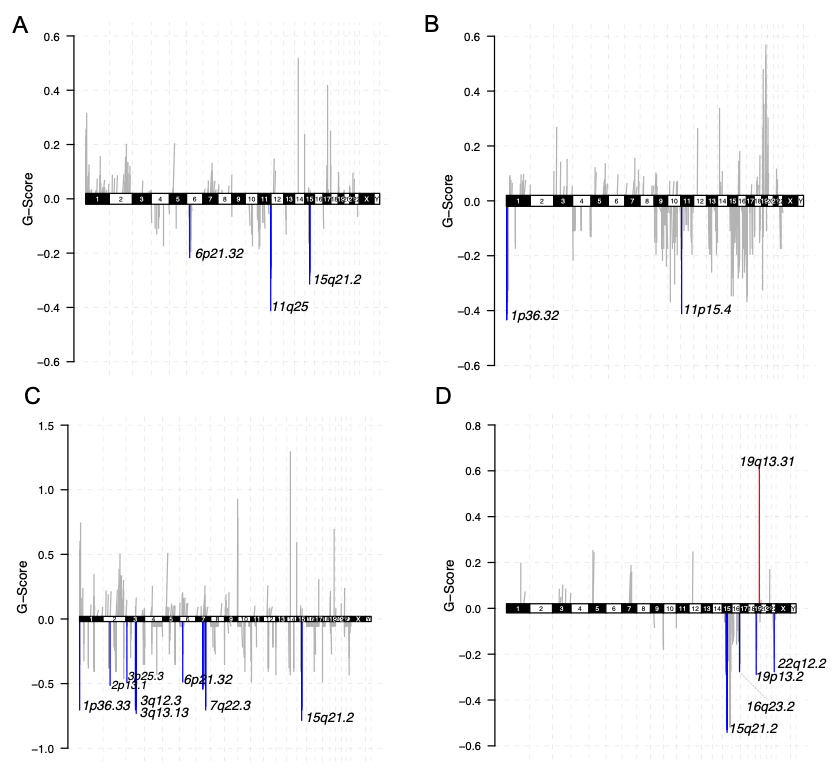


Supplementary Figure 3. Significant focal SCNAs in different patient groups. Colored bars indicate focal SCNAs with q < 0.15. Amplifications are depicted in red, while deletions are indicated in blue. (A) All NECCs; (B) All NECEs; (C) dMMR-like subtype of NECCs; (D) dMMR-like NECCs and NECEs.

1. **NECC1**


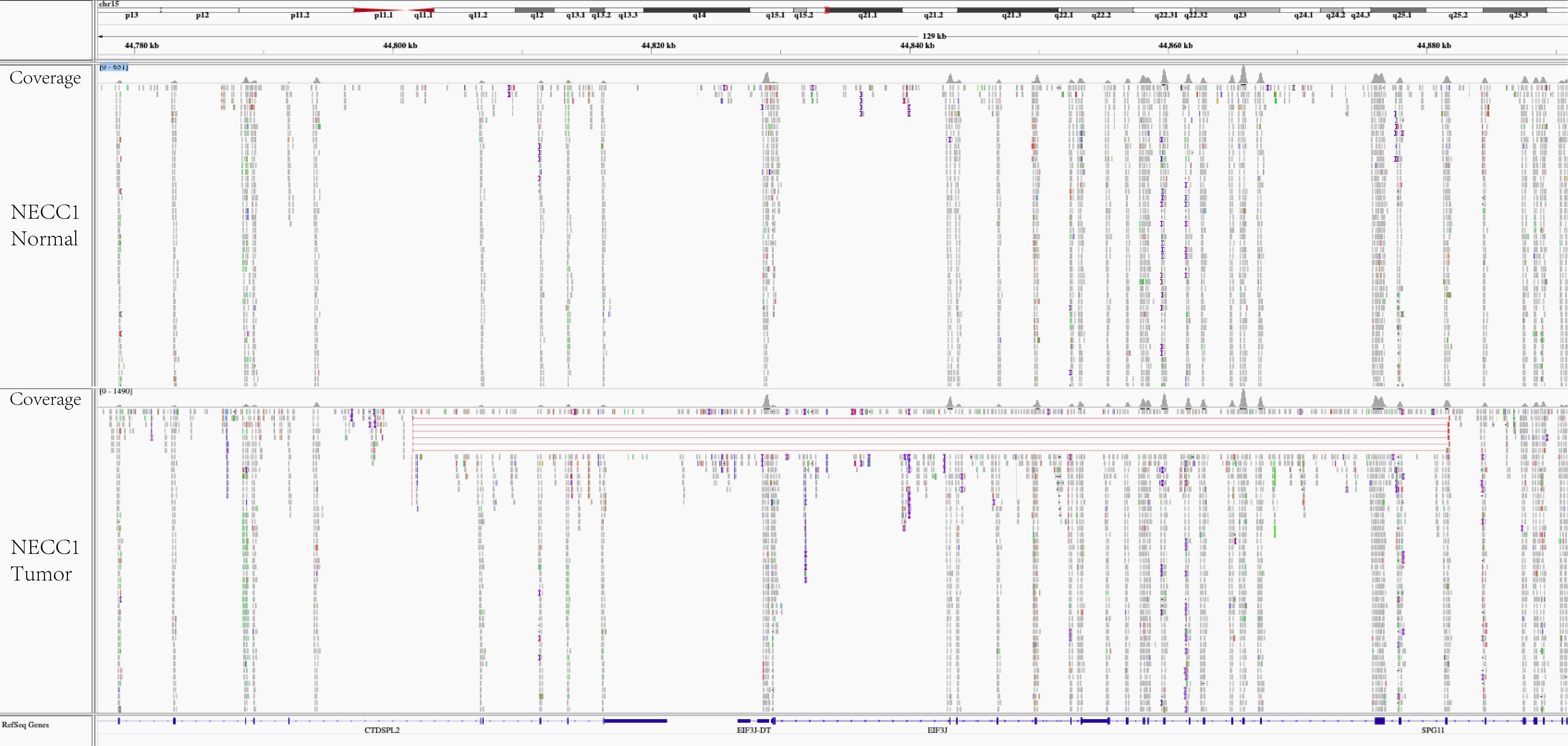


**(B) NECC3**


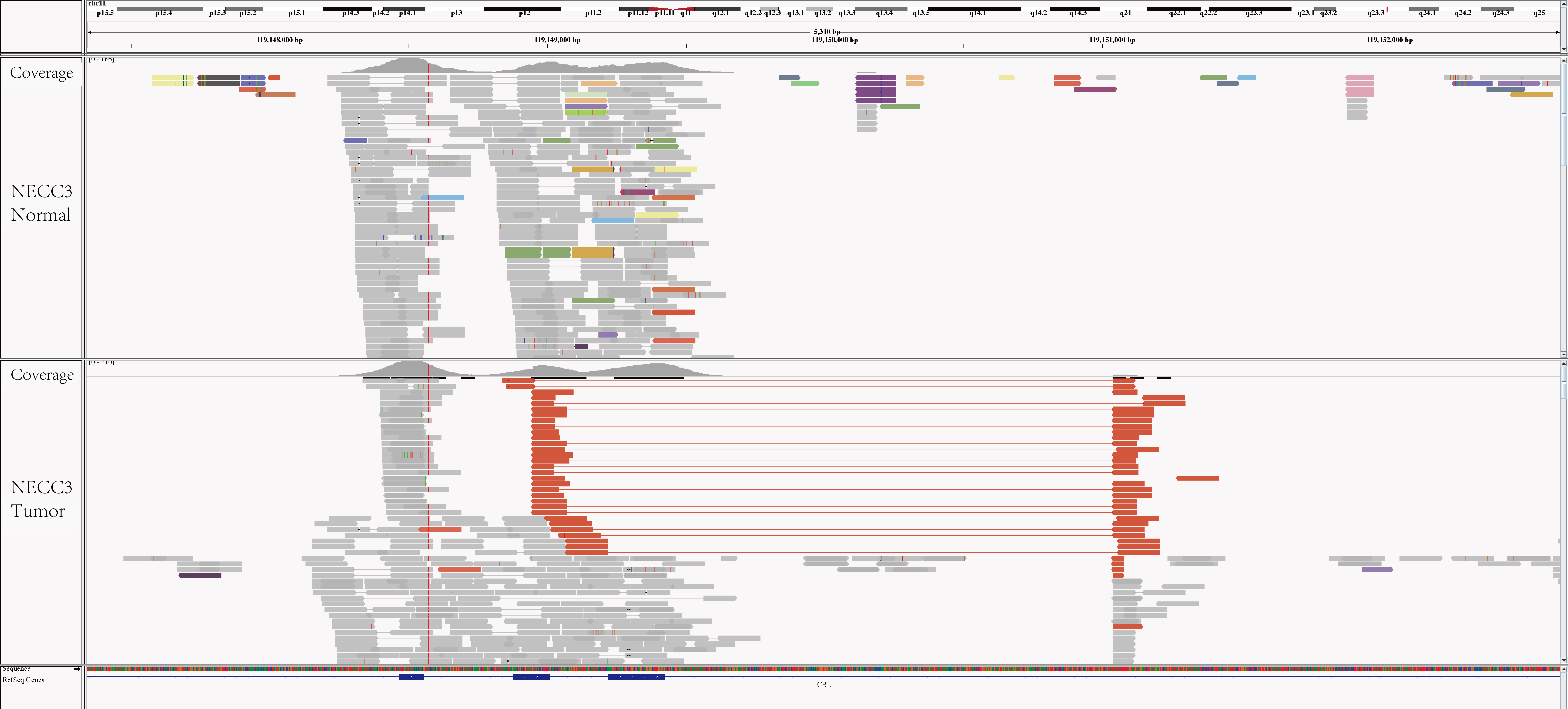


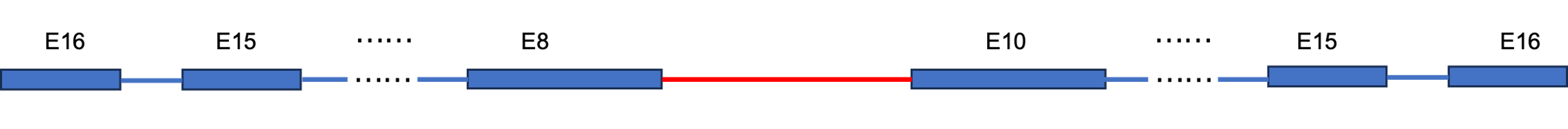


**(C) NECC4**


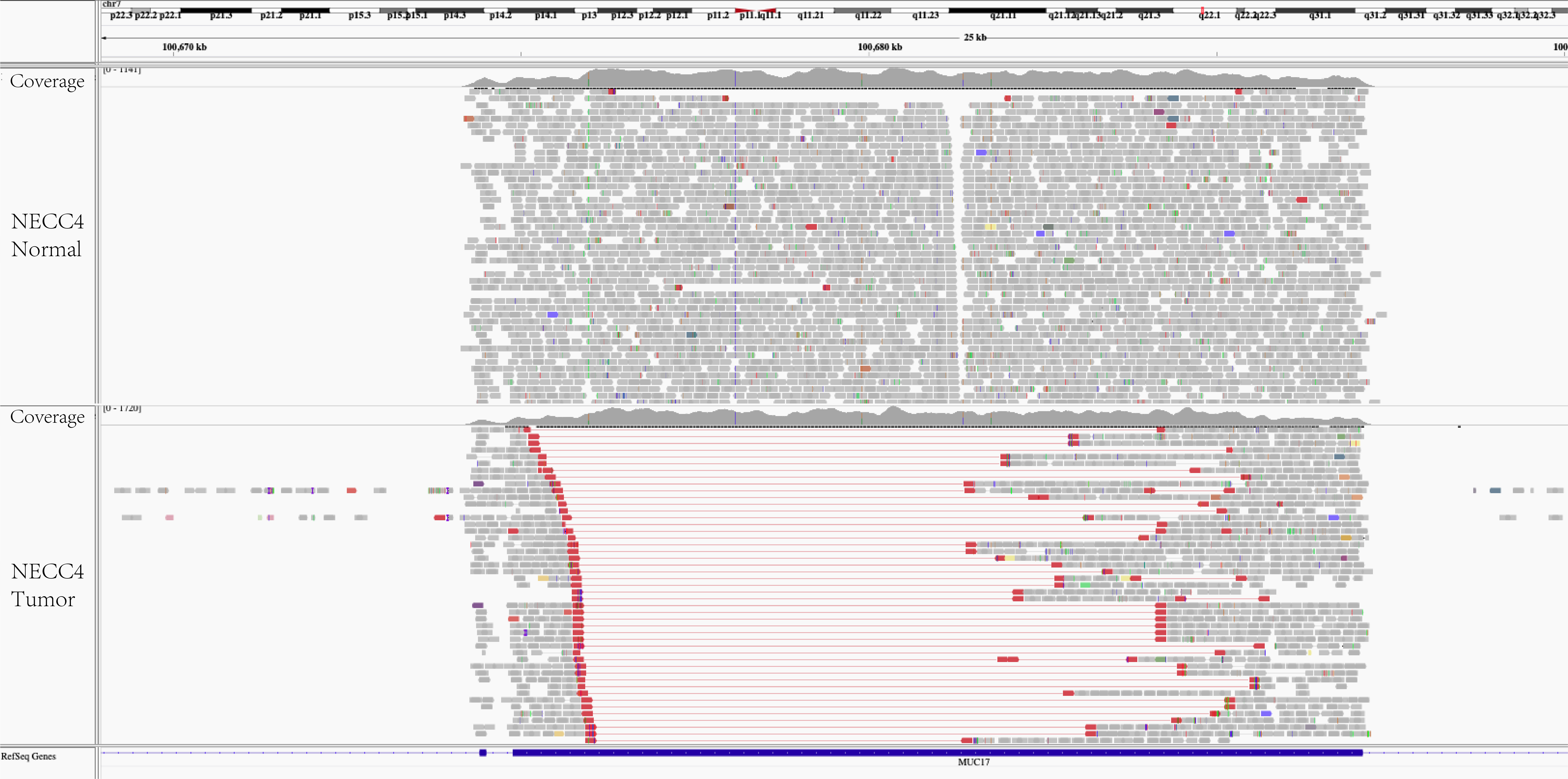


(**D) NECC5**


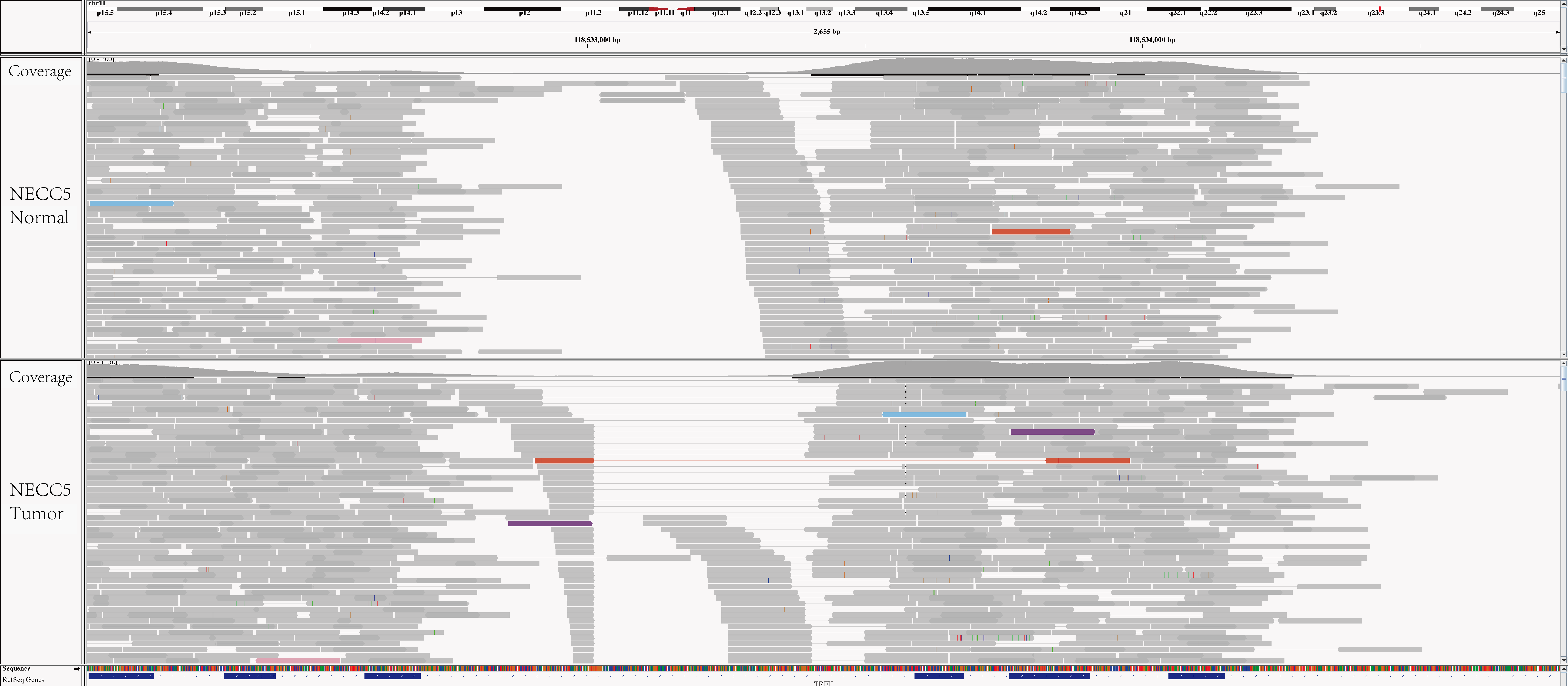


Supplementary Figure 4. IGV plots of SVs. The bottom of each panel shows the reads distribution of tumor samples, and top for its paired PBMC samples. Split reads that supporting the existence of SV were labeled by red. (A) Duplication of chr15:44,801,470-44,881,820 in NECC1. (B) Homology recombination of chr11:119,148,949-119,151,040 and its potential product in NECC3. (C) Deletion of chr7:100,681,223-100,681,722 in NECC4. (D) Deletion of chr11:118,533,010–118,533,381 in NECC5.

Supplementary Figure 5. Recurrent mutations in PI3K-AKT pathway and RAS gene family. Red font shows the mismatch repair deficiency type of NECC, blue font indicates micro-satellite instability type of NECE, and green font indicates the only NECC patients with APOBEC as the primary signature.
